# Interaction between Intestinal Parasites and the Gut Microbiota: Implications for the Intestinal Immune Response and Host Defence

**DOI:** 10.3390/pathogens13080608

**Published:** 2024-07-23

**Authors:** Jensine A. Grondin, Asif Jamal, Sadrina Mowna, Tyler Seto, Waliul I. Khan

**Affiliations:** 1 Farncombe Family Digestive Health Research Institute, McMaster University, Hamilton, ON L8S 4L8, Canada; grondij@mcmaster.ca (J.A.G.); jamala14@mcmaster.ca (A.J.); mownas@mcmaster.ca (S.M.); setot2@mcmaster.ca (T.S.); 2 Department of Pathology and Molecular Medicine, McMaster University, Hamilton, ON L8S 4L8, Canada

**Keywords:** host–parasite–microbiota axis, parasitic infection, intestinal parasites, helminths, protozoa, gut microbiota, parasitic defence

## Abstract

Intestinal parasites, including helminths and protozoa, account for a significant portion of the global health burden. The gastrointestinal (GI) tract not only serves as the stage for these parasitic infections but also as the residence for millions of microbes. As the intricacies of the GI microbial milieu continue to unfold, it is becoming increasingly apparent that the interactions between host, parasite, and resident microbes help dictate parasite survival and, ultimately, disease outcomes. Across both clinical and experimental models, intestinal parasites have been shown to impact microbial composition and diversity. Reciprocally, microbes can directly influence parasitic survival, colonization and expulsion. The gut microbiota can also indirectly impact parasites through the influence and manipulation of the host. Studying this host–parasite–microbiota axis may help bring about novel therapeutic strategies for intestinal parasitic infection as well as conditions such as inflammatory bowel disease (IBD). In this review, we explore the relationship between intestinal parasites, with a particular focus on common protozoa and helminths, and the gut microbiota, and how these interactions can influence the host defence and intestinal immune response. We will also explore the impact of this tripartite relationship in a clinical setting and its broader implications for human health.

## 1. Introduction

Intestinal parasitic infections have been shown to affect approximately 3.5 billion people worldwide and pose a significant health and economic burden on both a personal and global scale [1,2]. Helminths and protozoa represent a substantial portion of the parasitic infections that can impact the human gastrointestinal tract. These intestinal infections often result in malnutrition, severe diarrhoea, nausea and vomiting, and pose a significant health risk, especially in developing children. According to the World Health Organization, soil-transmitted helminth infections are estimated to affect 1.5 billion people globally, which accounts for approximately 24% of the world’s population [1]. Common helminth infections of the gut include ascariasis (*Ascaris lumbricoides*), trichuriasis (*Trichuris trichiura*), strongyloidiasis (*Strongyloides* sp.), enterobiasis (*Enterobius vermicularis*), and schistosomiasis (*Schistosoma* sp.) [1,3,4]. Major intestinally associated protozoan infections include cryptosporidiosis (*Cryptosporidium* sp.), cystoisosporiasis (*Cystoisospora belli*), cyclosporiasis (*Cyclospora cayetanensis*), balantidiasis (*Balantidium coli*), giardiasis (*Giardia lamblia*), and amebiasis (*Entamoeba histolytica*) [3,4]. 

Across evolutionary history, intestinal parasites have evolved tactics to utilize host resources, evade the host immune system, and establish an ecological niche. In turn, host immune strategies have coevolved increasingly sophisticated mechanisms of detection and immune pursual [5,6,7]. For successful parasitism to occur, invaders must be able to exploit the host, and a sufficiently susceptible host must provide an intestinal ecosystem in which the parasite can survive and thrive [8,9,10]. In this way, the conditions of the intestinal environment and the dynamic interactions between host and parasite play a significant role in determining pathogenic potential and, ultimately, disease outcome [6,7,8,9,10,11,12,13,14]. 

These host–parasite interactions do not occur in isolation and, thus, are preyto the broader ecological context of the gut, including the commensal microbiota [10]. Evidence suggests that the gut microbiota is a major mediator of the parasite–host relationship, not only through direct interactions with the parasitic organism but also by influencing the host immune response [5,10,15]. Every microbe in the gut ecosystem presents a new potential set of interactions in the host–parasite–microbe axis that may disrupt the delicate balance of homeostasis and influence parasitic pathogenicity [5,8,13,16,17]. Through pattern recognition receptors (PRRs) such as toll-like receptors (TLRs) and nucleotide-binding oligomerization domain (NOD)-like receptors (NLRs), the gut microbiota can interact directly with the host. These receptors sense pathogen or microbially associated molecular patterns (PAMPS or MAMPs) and help initiate downstream immunoregulatory mechanisms [8]. In turn, surveillance and immunological action by the host through agents such as antimicrobial peptides (AMPs), local immune cell populations, and mucus layer integrity controls and manipulates the microbial environment of the gut [18,19]. Immune function at the mucosal surface can also influence the pathogenic potential of intestinal parasites [8,19]. 

In relation to parasites, microbially derived products and direct competition from commensal microbes can shape clearance and infection severity [10,20,21,22]. In a reciprocal fashion, invading parasites can also influence local microbes by sequestering resources and through the direct action of parasite-produced antimicrobial agents such as excretory–secretory products (ESPs) and extracellular vesicles (ECVs) [23,24,25,26,27,28,29]. It should also be noted that the state of the gut microbiota can also play a major role in host damage incurred over the course of parasitic infection [8].

Though the multidirectional host–parasite–microbiota relationship is still under investigation, evidence increasingly suggests that these interactions have a major influence on host health and disease outcomes. Here, we aim to explore the tripartite relationship between host, parasite and microbiota in the context of the gastrointestinal (GI) tract and the implications of this relationship in the immune response and host defence, with a particular focus on protozoan and helminth infection. We will also explore the broad implications across clinical and animal models and how these implications may shape public health.

## 2. The Impact of Intestinal Parasites on the Gut Microbiota 

In the complex microcosm of the gut, the commensal microbiota poses an obstacle to the establishment and survival of invading helminths and protozoa. The gut microbiota is essential to the development of a functional host immune system [30] and also helps to provide direct competition and protection from pathogenic organisms [31,32,33]. Thus, strategies employed by parasites themselves, either directly on microbes or indirectly via manipulation of the host, play a role in the establishment of infection. In both clinical and animal models, the function and composition of the gut microbiota have been shown to be altered in parasitic infection [34,35,36,37,38,39,40,41,42,43]. 

Microbial changes induced by intestinal parasites have been well documented, particularly in murine models. For instance, infection with the murine helminth *Trichuris muris* was shown to drive changes in microbial composition and α- and β-diversity in mice on both days 14 and 28 post-infection [34]. These changes were characterized by a reduction in the abundance of the Bacteroidetes taxa, specifically *Prevotella* and *Parabacteroides*. Moreover, 91 days following *T. muris* infection, the microbiome gradually reverted to a composition similar to that of an uninfected mouse, indicating that the altered microbiota composition is tied to infection status. Holm and colleagues (2015) found similar results, which identified that chronic *T. muris* infection altered the microbiome composition in a way that decreased the microbial diversity and increased the relative abundance of bacteria from the *Lactobacillus* genus [36]. Furthermore, in mice administered the parasitic helminth *Heligmosomoides polygyrus*, a significant shift in both the relative distribution and abundance of intestinal bacteria were present in infected mice. Specifically, bacteria from the *Lactobacillaceae* family were significantly increased in the murine ileum [37]. Mice infected with *Trichinella spiralis* also show an altered gut microbiome profile compared to controls, with a notable increase in the relative abundance of Proteobacteria and a reduction in Bacteroidetes and Clostridiales [38,39]. In addition, Barash et al. (2017) identified that mice infected with the protozoan *Giardia* had altered microbial diversity and an increase in aerobic Proteobacteria as well as decreases in anaerobic Firmicutes and Melainabacteria in both the hindgut and foregut [40]. Together, these findings indicate that both helminth and protozoan parasitic infections can alter the gut microbiome in murine models. 

In humans, alterations in microbial composition with helminth and protozoan infection have also been reported. In rural Malaysia, those colonized with helminth parasites displayed greater microbial species richness and increased abundance of *Paraprevotellaceae* [41]. In a Sri Lankan population, beta diversity was significantly increased, while alpha diversity was unaffected in nematode-infected individuals when compared to uninfected individuals. In infected individuals, *Verrucomicrobiaceae* and *Enterobacteriaceae* were spiked compared to uninfected controls [42]. A study of over 1200 children in Guinea-Bissau, however, reported that helminth infection did not alter microbial composition, but for those infected with protozoa, including *Entamoeba* and *Giardia*, the overall composition was significantly affected [35]. Work conducted in Southwest Cameroon also showed that the presence of *Entamoeba* was significantly correlated with changes in microbial composition and diversity regardless of factors including sex, age, body mass index, ancestry, or location [43]. Though these findings lend support to the idea that the presence of helminths and protozoa influence microbiota composition in human populations, regional and environmental factors, as well as the starting microbial composition prior to infection, must be taken into account. Many regional studies exploring gut microbiota changes in the presence of parasitic infection in human populations are conducted with smaller sample sizes, and direct correlations between specific parasites and microbial changes are often not reported. With that being said, more work needs to be done exploring how helminth and protozoan infections shape the microbiota in human populations.

Though the mechanism of action by which helminths can influence the microbiota is still under investigation, the release of ESPs by these worms has been shown to have microbiota-modulating effects. ESPs released by helminths participate in several biological processes, including aiding in evasion as well as acting as immunomodulators and as communicatory signals between individual parasites [44,45,46,47]. These ESPs come in various forms, such as immunomodulatory proteins, glycoproteins, and small RNAs [48,49]. Intriguingly, evidence suggests that these ESPs have antimicrobial activity as well [23,24,25]. In this way, helminths can directly impact microbial composition and influence the survival and function of various microbes [23,24,25,26,27,50]. For instance, recent work by Rooney et al. (2022, 2024) found that ESPs released by the helminth *Teladorsagia circumcincta* impacted *Escherichia coli* [28] and *Bacillus subtilis* [29] growth and survival in vitro. Evidence suggests that helminths and helminth-derived products can also influence host PRR signalling and alter the expression patterns of TLRs to regulate host recognition of microbes [51,52,53,54]. In addition, helminths can also modulate the responsiveness of immune cells to TLR signalling [55]. The release of ECVs from helminths may also heavily impact microbiome interactions through antimicrobial properties and host immunomodulation [56].

**Figure 1:**
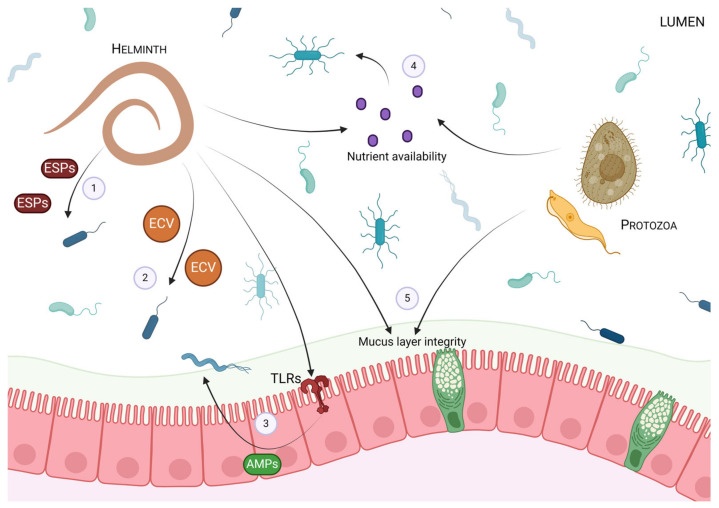
Overview of the impact that protozoan and helminthic infection can have on the gut microbiota. Protozoan and helminth intestinal infections have been reported to induce changes in microbial composition and diversity in both human and animal models. Helminths have been shown to impact the gut microbiota in a variety of ways, including (1) the release of excretory–secretory products (ESPs), (2) the release of extracellular vesicles (ECVs), and (3) modulating the release of host-derived antimicrobial peptides (AMPs) through toll-like receptors (TLRs). Both protozoan and helminth infection in the gut can (4) impact nutrient availability and (5) mucus barrier integrity, and thus, indirectly affect the microbes of the gut.

Additionally, helminth colonization can affect the nutrient and niche availability in the GI tract. For example, helminth infection can impair epithelial glucose absorption and promote an environment favouring the growth of certain bacteria [23]. Furthermore, altered mucus production and goblet cell function, which are hallmarks of several intestinal helminth infections, can affect the nutrient availability and habitat configuration for certain microbes, particularly mucus residers [23]. Some species of parasitic protozoa, including *E. histolytica*, *Giardia intestinalis*, and *Tritrichomonas suis*, are also able to alter intestinal mucus abundance and composition by the production of mucolytic enzymes [57,58], which may ultimately affect the survival of mucophilic bacteria. An overview of the impact that protozoan and helminthic parasites can have on the microbiota is presented in Figure 1.

## 3. The Impact of the Gut Microbiota on Intestinal Parasites 

Interactions between intestinal parasites and the gut microbiota are not unidirectional; the gut microbiota can directly influence parasitic establishment, survival, colonization, and expulsion in the host. Studies conducted in germ-free (GF) mice, which lack a microbiota, exemplify the impact that the presence of microbes has on parasitic infection. In GF mice, the helminth *H. polygyrus* has been found to have altered gene expression compared to worms colonizing specific-pathogen free (SPF) mice, suggesting that the presence and/or absence of the gut microbiota can manipulate parasitic gene expression patterns [24]. These changes in the gene expression patterns were correlated with reduced worm fitness in GF mice, highlighting the contextual impact of the gut microbiota on infection [24]. 

The microbial load can also impact the establishment of parasitic infection. In vitro work by Hayes et al. (2010) elucidated that mouse cecum explants, as well as the isolated bacterial strains *E. coli*, *Pseudomonas aeruginosa*, *Staphylococcus aureus*, and *Salmonella typhimurium* and the yeast, *Saccharomyces cerevisiae*, induced *T. muris* egg hatching and that direct contact between bacteria and egg is required for hatching to occur [59]. The authors suggested that this phenomenon is partially mediated, in a species-specific manner, by bacterial adherence via type 1 fimbriae [59]. Furthermore, in vivo work demonstrated that in mice treated with the antibiotic enrofloxacin, the establishment of *T. muris* infection was disrupted, and mice administered these antibiotics showed a diminished worm burden 21 days post-infection [59]. Taken together, these findings suggest that the establishment of *T. muris* is dependent on the presence and direct signalling of the microbiota in the gut. 

Not only does the presence or absence of the microbiota play a key role in parasitic fitness and establishment but the ability of these organisms to manipulate the intestinal environment can impact infection as well. Local microbes can impact parasites and the intestinal environment by sequestering essential nutrients and through the production of microbially derived products. Funkhouser-Jones et al. (2023) showed that several microbially derived metabolites have inhibitory effects on the growth of *Cryptosporidium parvum*. In particular, indoles seem to impact the growth and intracellular lifecycle of this protozoan by modulating host mitochondrial function, promoting endoplasm reticulum stress and impairing the function of the parasitic mitosome. These results were reflected in vivo; mice supplemented with indoles or indole-producing bacteria displayed improved *C. parvum* infection resistance [60]. 

Intriguingly, the gut microbiota also has a role in adjuvant therapies aimed at promoting helminth expulsion. For instance, β-glucans, a class of polysaccharides, have been shown to trigger gut microbiota-dependent expulsion of particular helminths [61]. Indeed, the β-glucan, lentinan (LNT), can serve as an effective adjuvant to the Ts-Serpin vaccine against *T. spiralis* [39]. It should be noted that the gut microbiota is both altered by and has a role in the LNT-induced host defence against this helminth [38]. When *T. spiralis*-infected mice are treated with LNT, infection-induced changes in Bacteroidetes and Clostridiales can be restored to levels found in the control group. Furthermore, *T. spiralis*-infected mice treated with both LNT and antibiotics displayed significantly higher worm burdens than infected mice treated with LNT alone. These findings suggest that the gut microbiota has an essential role in the effectiveness of LNT as an adjuvant against *T. spiralis* infections. 

Investigations concerning the influence of the gut microbiota on either parasitic colonization or expulsion are not limited to murine models; avian models have also illustrated this phenomenon. Obligate intracellular parasites belonging to the genus *Eimeria*, which most commonly infect chickens [62], disrupt intestinal homeostasis by invading and rupturing intestinal epithelial cells, leading to enteritis and, in some cases, hemorrhage [63]. Of the seven species of *Eimeria*, *Eimeria tenalla* has been reported to be the most virulent and is responsible for high levels of morbidity and mortality in poultry flocks [62]. Work by Gaboriaud et al. (2020) has investigated whether the gut microbiota influences *E. tenalla* infection and clearance in chickens. In animals inoculated with different doses of *E. tenalla* oocytes, GF chickens displayed significantly reduced cecal oocyte load at days 6, 7, and 9 post-infection compared to conventionally raised chickens [62]. Furthermore, the gut microbiota was shown to impact the development and growth of *E. tenalla*, with fewer and less developed first- and second-generation schizonts present in GF chickens compared to conventional animals [62]. These findings collectively suggest that the growth, development, and survival of the parasite *E. tenalla* in the intestinal tract of chickens is dependent on the presence of the gut microbiota.

In humans, common protozoan infections, including *Entamoeba*, *Giardia*, *Cryptosporidium*, and *Blastocystis* sp., which mainly localize within the intestinal mucosa, interact with the surrounding gut microbiota [64,65]. Not only do these parasites influence the microbial composition in humans but the gut microbiota itself can influence the colonization, survival, and pathogenicity of these protozoans. A recent study conducted in humans examined the gut microbiota profile of asymptomatic and symptomatic individuals with and without the intestinal protozoa *Blastocystis* sp. [66]. It was found that regardless of symptomatology, the presence of *Blastocystis* sp. *ST3* led to significant alterations in the gut microbiota profile, with greater species diversity in asymptomatic individuals. In addition, culturing *Blastocystis* sp. from symptomatic individuals in conjunction with bacteria from asymptomatic individuals, in vitro, led to a significant increase in the parasite number and a slight increase in protease activity [66]. Conversely, culturing *Blastocystis* sp. from asymptomatic individuals with bacteria from symptomatic individuals was correlated with a decrease in the parasite number as well as a significant increase in protease activity and colonic cell proliferation. Taken together, these findings emphasize the influence of the gut microbiota on *Blastocystis* sp. growth and pathogenicity.

Despite multiple studies demonstrating a key role for the gut microbiota in either helminth colonization or clearance, it should be noted that there are instances when commensal bacteria do not affect the expulsion of intestinal parasites. *Hymenolepis diminuta* is an example of one such parasite that can be cleared independent of the gut microbiota [67]. This tapeworm commonly infects rodents and serves as an important model organism for studying cestodiasis [68]. It is well established that the expulsion of *H. diminuta* in mice is dependent on signal transducer and activator of transcription (STAT)-6 signaling [69]. However, recent work by Shute et al. (2020) has explored the idea that the expulsion of this parasite is not dependent on the gut microbiota. Wild-type BALB/c and C57BL/6 mice infected with *H. diminuta* typically expel the worm burden in 9 to 12 days post-infection [67]. Notably, treating infected mice with broad-spectrum antibiotics did not significantly alter the expulsion of *H. diminuta*, goblet cell hyperplasia, or eosinophilia. Further investigations using GF mice demonstrate that, as in SPF mice, *H. diminuta* is cleared by approximately day 10 post-infection.

**Figure 2:**
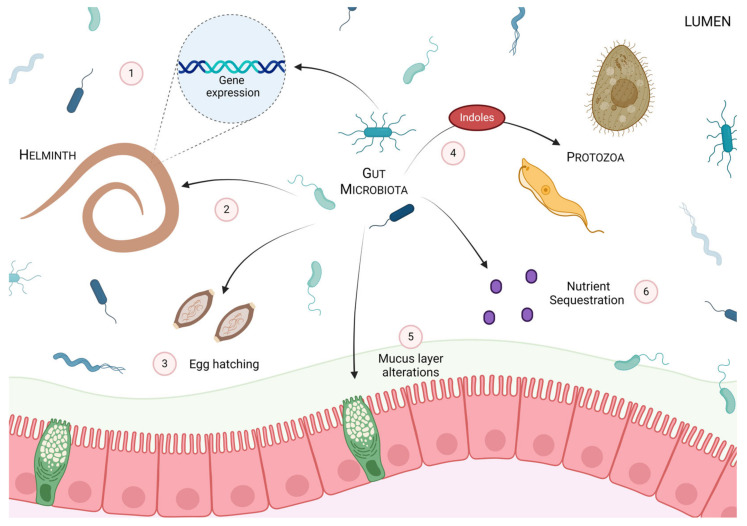
Summary of the potential ways the gut microbiota can impact parasite fitness and infectivity. The gut microbiota can both directly and indirectly impact intestinal parasite establishment, survival, colonization and expulsion within the host. Microbes have been shown to (1) induce altered gene expression in helminths, (2) directly impact helminth fitness, and (3) promote helminth egg hatching. The microbiota can also (4) inhibit the growth of particular protozoa through the production of metabolites such as indoles. Further, microbes can influence the overall environment of the gut by (5) impacting mucus layer integrity and (6) sequestering nutrients, both of which impact parasite fitness.

The aforementioned research illustrates the significance of the gut microbiota in both helminth and protozoan survival and fitness, as well as disease outcomes. Notably, however, despite their coevolution in the gut ecosystem, microbial-independent mechanisms for parasite clearance do occur, and clearance in these instances is seemingly more dependent on host immune function. A summary of the effects of the gut microbiota on parasitic fitness is found in Figure 2.

## 4. Modulation of Host Immune Responses and Parasitic Defence via Microbial Manipulation 

In addition to the direct effects of the gut microbiota on certain parasitic infections discussed so far, multiple studies have demonstrated that the gut microbiota can influence parasitic clearance indirectly through modulation of the host immune response and host defensive adaptations. 

The impact of the microbiota, via host immune modulation, on parasitic infection is neatly illustrated in instances where the influence of these microbes is absent. Reynolds et al. (2014) highlighted this phenomenon with work utilizing *H. polygyrus*. When infected with this helminth, GF mice, which lack a gut microbiota, displayed altered regulatory T cell (Tregs) populations and an altered Th2/Treg ratio in the small intestine compared to SPF controls. Under normal infection conditions, *H. polygyrus* promotes the activation of Tregs in order to suppress the host immune response, including host-protective Th2. Further, in conventionally raised susceptible mice, the presence of *Lactobacillus* was altered with the administration of *H. polygyrus*, a change that was not found in mice able to clear the infection [70]. Intriguingly, supplementation with the commensal *Lactobacillus taiwanensis* in resistant mice promoted *H. polygyrus* infection, likely through the expansion of the aforementioned Treg populations. Rausch et al. (2018) also paralleled these findings in GF mice and found that in the absence of a gut microbiota, the ratio of Th2/Treg was increased, a factor that may account for the decreased parasite fitness found in these mice [24]. Work by Su et al. (2017) has also shown that *H. polygyrus* infection can significantly alter microbial composition, with a marked increase in the abundance of Bacteroidetes and decreases in Firmicutes and Lactobacillales [71]. When transferred to antibiotic-treated recipient mice, this altered microbiota increased susceptibility and exacerbated colitis associated with the bacterial pathogen *Citrobacter rodentium* [71]. This effect was ascribed to a shift in the levels of IL-10 and Tregs [71], resulting in a dysregulated antimicrobial response. Additionally, infection with *H. polygyrus bakeri* in GF mice resulted in an increased worm burden and altered parasitic distribution due to changes in intestinal motility attributed to the absence of the microbiota [72]. These results, taken as a whole, suggest that by influencing the host immune system, the gut microbiota can alter parasitic infection susceptibility.

The gut microbiota can also mediate host-derived physical changes in the intestinal environment, particularly those in the mucus layer, which can aid in parasitic expulsion. One such example is the effect of microbial species on the establishment of the parasitic roundworm *T. spiralis* [38]. In humans, *T. spiralis* infection mainly occurs through the consumption of raw or undercooked pork and is considered a major global health burden [73,74]. The successful expulsion of this helminth during the early stages of infection is mediated through host goblet cell hyperplasia and increased mucin production [75]. By influencing mucus, the gut microbiota can sway the host’s ability to expel *T. spiralis*; administration of the butyrate-producing bacteria *Clostridium tyrobutyricum* has been shown to increase mucin secretion and the number of goblet cells, and to significantly reduce *T. spiralis* worm burden in C57BL/6J mice [38]. Previous work from our lab has shown that in *T. muris* infection, treatment with the bacteria *Lactobacillus rhamnosus JB-1* influences host defence, enhances worm expulsion and modulates goblet cell biology via host IL-10 production [76]. We have also previously reported the importance of the microbiome-sensing intracellular NOD proteins in shaping the mucus layer and altering host defence against *T. muris* infection [77]. In our work, mice deficient in both NOD1 and NOD2 displayed significantly diminished goblet cell numbers and MUC2 expression, key contributors to parasite clearance. Lower numbers of goblet cells and MUC2 were also found in GF mice, and treatment with NOD1 and NOD2 agonists recovered this effect, emphasizing the role of the microbiota in host innate defence and clearance in intestinal parasitic infection [77]. 

The mucus layer also serves as a source of nutrition for the protozoan parasite *E. histolytica*. Infection with this parasite can result in amebic colitis and liver abscesses by penetrating the mucus layer and intestinal mucosa. *E. histolytica* can also feed on and interact with resident microbes, directly impacting commensals such as *Faecalibacterium prausnitzii* and *Bifidobacterium longum* [78]. The severity of infection with *E. histolytica* has been found to be attenuated by the presence or absence of the microbiome. In both *Muc2^−/−^* and *Muc2^+/+^* mice treated with antibiotics, *E. histolytica* induced heavy mucus secretions and upregulated the proinflammatory cytokines IFN-γ and TNF-α compared to nonantibiotic-treated mice; however, fecal transplants in these antibiotic-treated mice attenuated this parasitic induced hyperactive host response [79]. These findings emphasize that pathogenicity and normal host defensive responses are often dependent on the presence of a healthy, intact colonic microbiota, as GF mice are unable to produce a robust innate defensive response to infection [78]. The relationship between *E. histolytica*, the host immune response and the microbiota has been extensively covered in a review by Leon-Coria et al. (2020) [78].

In the intestinal environment, secreted AMPs serve as a key component of pathogenic defence. Despite the vast array of AMPs, nearly all function to kill or inactivate microorganisms by targeting their cell wall or membranes and, ultimately, prevent them from interacting with and invading the underlying epithelium [80,81]. A recent study has demonstrated the antimicrobial properties of a unique host-derived bactericidal protein, small proline-rich protein 2A (SPRR2A), in the context of helminth infection [82]. SPRR2A expression is upregulated in response to various helminth infections, including *T. spiralis*, *Nippostrongylus brasiliensis*, and *H. polygyrus*. This upregulation was found to be mediated by type 2 cytokines such as IL-4 and IL-13, which play a vital role in helminth clearance. The gut microbiota can also influence SPRR2A expression; in conventionally raised mice, *Bacteroides thetaiotaomicron* administration increased SPRR2A expression to varying magnitudes. This microbiota-induced upregulation of SPRR2A expression was found to be mediated via the TLR-MyD88 signalling pathway. Reciprocally, SPRR2A itself alters the gut microbiota composition and prevents the colonization of certain bacterial species; SPRR2A-deficient mice (*Sprr2a^−/−^*) exhibited an increased relative abundance of Gram-positive bacteria in the small intestine compared to wild-type mice, with a notable increase in *Lactobacillus*, *Turicibacter*, and *Candidatus Arthromitus*. Furthermore, *H. polygyrus*-infected *Sprr2a^−/−^* mice harboured a greater bacterial load within intestinal tissue compared to wild-type mice, suggesting a key role for SPRR2A in preventing bacterial invasion during helminth infection. Overall, these findings illustrate that SPRR2A is both influenced by and interacts with the intestinal microbiota and modulates host defence capabilities during helminth infections. 

It should also be noted that in certain contexts, helminth infections provide beneficial stimulation of the host’s immune responses and gut microbiota. This is exhibited in NOD2-knockout mice chronically infected with *T. muris* or *H. polygyrus* [83]. As touched on above, NOD2 is mainly a bacterial sensor, which triggers inflammatory and antimicrobial gene expression in response to the muramyl dipeptide present in the cell walls of both Gram-negative and Gram-positive bacteria [84]. Mutations in NOD2 are associated with chronic inflammation, defects in goblet cell morphology and function, and the onset and progression of Crohn’s disease [85]. However, these abnormalities seemed to be reversed in NOD2^−/−^ mice infected with either *T. muris* or *H. polygyrus*. These mice exhibit a restoration of goblet cell number and morphology, as well as reduced inflammatory markers, including antimicrobial lectin, RegIII-β, and interferon (IFN)-γ+ CD8+ intraepithelial lymphocytes [83]. Changes in the gut microbiota may be responsible for these effects as both helminths reduced the *Bacteroides vulgatus* levels, which are characteristically elevated in the small intestine of NOD2^−/−^ mice.

**Figure 3:**
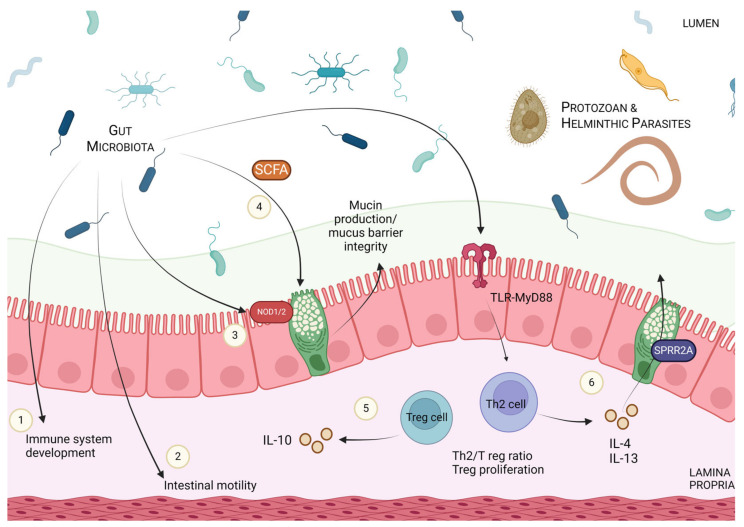
Overview of the various ways microbial manipulation can modulate host immune responses and parasitic defence. In addition to the direct manipulation of the luminal environment, the gut microbiota can also indirectly impact parasitic fitness and clearance through interactions with the host. These include, but are not limited to, (1) impacting the development of the immune system, (2) altering intestinal motility, impacting the mucus barrier integrity and mucin production through (3) NOD1/2 signalling and the production of (4) short-chain fatty acids (SCFAs). Microbes can also impact (5) the ratios of Th2/Treg cells, Treg proliferation and the production of the anti-inflammatory cytokine, IL-10. (6) The gut microbiota has also been shown to, through the TLR-MyD88 pathway, alter the production of IL-4 and IL-13 and, subsequently, the release of antimicrobial peptides like SPRR2A, key factors in parasite clearance.

Taken as a whole, the research discussed above emphasizes how, via microbial manipulation, modulation of the host immune responses and the intestinal environment can greatly impact parasitic infection outcomes (summarized in Figure 3). Though the exploration of this topic is still in its infancy, and more work needs to be done, the tripartite relationship between host–microbiota–parasite has clear implications in the realm of public health.

## 5. Clinical Implications of the Host–Parasite–Microbiota Axis

### 5.1. Microbial Manipulation in Parasitic Infection

On a global scale, intestinal helminths and protozoa account for millions of infections every year, particularly in the developing world [1,3,4]. The consequences of these infections including, malnutrition, growth stunting, and dehydration, pose significant health risks and greatly impact quality of life [1,3,4]. Both helminth and protozoan intestinal parasites, including *Ascaris*, *Entamoeba*, *Toxoplasma*, *Cyclospora*, *Giardia*, and *Cryptosporidium*, are among the top contributors to the global disease burden [3,4,86]. Thus, controlling and treating these infections is of great importance to public health. 

Current public health measures, including encouraging handwashing and sanitary food preparation practices, and providing access to clean water to interrupt transmission, have been at the forefront of controlling parasitic infection [3,4,87]. On a population level, public health policies and practices influence the evolutionary arms race between parasite and host, and shape the gut microbiota [8]. Chemotherapeutic strategies, such as the popular antihelminth drug praziquantel, have also been deployed to rapidly decrease individual parasite load and control transmission [3,88,89,90,91]. Mass drug administration (MDA) can be deployed in areas where helminth or protozoan infections are common; however, this strategy is expensive to administer, provides only a short-term solution, and has an efficacy that often does not span different parasitic species [87]. These chemotherapeutic strategies also pose a major risk of drug resistance, reinfection post-treatment, and issues with longer-term control [3,88,89,90]. Thus, developing alternative strategies is crucial and studying the relationship between host, parasite, and microbiota may give rise to novel treatment options for those with parasitic infection. 

Capitalizing on the direct or indirect effects of microbial manipulation on intestinal parasites may be one such strategy. Indeed, administration of the probiotic bacterial strains *Lactobacillus casei* and *Enterococcus faecium* has yielded encouraging results in controlling amoebiasis (*E. histolytica*). A significant reduction in parasitic survival, particularly when these microbes are used in combination, has been reported; in vitro work suggests that the administration of these probiotics in coculture with *Entamoeba* can affect growth, proliferation, and, ultimately, survival [92]. In animal models, *L. casei* and *L. rhamnosus* have also proven beneficial in the clearance of *Giardia* [93,94]. Probiotics may also help increase the efficacity of traditional drugs; clinical studies have shown that administration of the yeast *Saccharomyces boulardii* in combination with metronidazole, a popular anti-bacterial and anti-protozoan drug, is more efficacious than metronidazole alone across multiple types of protozoan infection [95,96,97]. In addition to probiotics, the impact antibiotics may indirectly have on the intestinal environment via promoting microbial dysbiosis is also under consideration for controlling both protozoan and helminth infection [78]. Reintroducing lost “old friends” or heirloom species with known immunoregulatory properties [17,98], nutritional interventions aimed at altering the microbiota [99], and supplementing with post-biotics or bacterially derived products, such as SCFA, may all provide exciting new avenues of research.

Manipulating the microbiota present in nematodes themselves may also prove an interesting therapeutic approach; targeting microbes that specifically colonize nematodes may disrupt worm development and survival and, subsequently, impact infectivity and disease outcomes [99]. Strategies such as the utilization of specific bacteriophages to target these microbes may be an option for future treatment and prevention [100]. 

Overall, probiotics and other microbial manipulating strategies may provide alternative or supplemental ways of combating intestinal parasitic infection. Probiotics can modulate host immunity, increase competition in the gut, shape intestinal niches and, significantly, mitigate the ever-building drug resistance promoted by traditional chemotherapeutic drugs [3,10,101]. 

### 5.2. Utilization of Intestinal Parasites in Immunoregulatory Conditions 

Suppressing the host immune system is an important survival strategy for parasites. As “old friends” of the mammalian gut, helminths may have helped blunt overzealous immune reactions and may have provided essential immunoregulatory priming for the host [17]. When these immune-regulating organisms are no longer active participants in the gut, as in modern industrialized society, unchecked and persistent inflammation may be a consequence [17,98,101]. In this way, the eradication of helminth infections may have implications for autoimmune diabetes, allergies and other immunologically driven conditions, like inflammatory bowel disease (IBD) [17,98,101,102,103,104,105,106].

IBD is an umbrella term for conditions characterized by chronic inflammation of the GI tract, including ulcerative colitis (UC) and Crohn’s disease (CD). Though the cause of and cure for these conditions are presently unknown, current thinking suggests a mix of genetics, environmental factors, dysregulated immune responses and, increasingly, alterations in the gut microbiota to contribute to the pathogenesis [107,108].

As the incidence of IBD increases worldwide, particularly in Westernized countries [109,110], treatment with helminths has been increasingly explored as an alternative therapeutic option. Exploiting the immunoregulatory properties of helminths and helminth-derived products may provide a way to balance the overactive and inappropriate immune responses prevalent in IBD [111]. Indeed, in animal models, treatment with helminth species has supported potential anti-colitic effects [111]. For instance, *T. spiralis* infection prior to the administration of dinitrobenzene sulfonic acid (DNBS) conferred protective benefits in this model of colitis by manipulating the balance of Th1 and Th2 cytokines [112]. Further, mice administered antigens from this same parasite displayed minimized effects of DNBS-induced intestinal pathology, which was associated with downregulated levels of IL-1β and upregulated IL-13 [113]. These findings support the notion that helminth and helminth-derived products, at least in part, work by rebalancing the immunological milieu in persistent intestinal inflammation. In humans, early clinical trials utilizing *Trichuris suis* yielded promising results as a potential therapy for both UC and CD [114,115]. Since then, small-scale clinical trials have also reported some success with helminth-based therapies [111,116]; however, a new emphasis on microbial considerations must be taken into account.

Recent work by Shute et al. (2021) has emphasized the idea that the gut microbiota plays a role in conferring protective benefits from helminths in IBD-like states. Shute et al. reported that mice infected with the tapeworm *H. diminuta* and administered DNBS to induce colitis showed ameliorated colitis severity in comparison to controls [117]. Intriguingly, the protection conferred by *H. diminuta* was dependent on the presence of the microbiota, as this protection was not seen in antibiotic-treated or GF mice. When treated with bacterial filtrates from mice infected with *H. diminuta* that were high in the SCFAs, acetate and butyrate, recipient mice were also protected from colitis. The authors showed that these microbial changes helped confer protection by influencing the anti-inflammatory cytokine IL-10. These findings emphasize the importance of microbial composition and function as a consideration when utilizing helminths in the context of IBD. It should be noted that, despite this work, the role the microbiota plays in helminth-derived protection in inflammatory bowel conditions has not been extensively studied, and further research investigating whether the anti-colitic effects conferred from helminth administration are exclusive to the helminth themselves or are derived from helminth-induced microbial changes is warranted. 

Thus, the use of particular helminths or helminth-derived products may prove beneficial by restoring balance in the dysregulated immune environment characteristic of IBD, allergies [118], and potentially, metabolic conditions like type 2 diabetes [119]. Research suggests that these benefits may be, at least in part, mediated through the intestinal microbiota. Though the details of these potential treatments are beyond the scope of this review, an extensive overview of helminth-based therapies in experimental colitis and IBD can be found in the recent papers by Arai et al. (2022) and Shields et al. (2022) [111,116].

## 6. Conclusions and Future Directions

Globally, intestinal helminth and protozoan infections are responsible for significant economic and personal burdens and disproportionality affect those in the developing world. Over an intertwined evolutionary history, interactions between parasite and host have undoubtedly shaped the biology of both organisms. However, these interactions do not occur in isolation. In the context of the gut, bacteria and other microbes influence both the host and invading parasites. In turn, both the host and parasite can shape the gut microbiota. Studying this multidirectional relationship not only deepens our understanding of the dynamics of parasitic infection but may also, in the future, aid in the development of microbially focused treatments. Considering the impact of intestinal parasites on livestock, companion animals and ecosystems, these treatments may have a major impact on the global economic burden brought about by parasitic infection. In conjunction with public health measures and current chemotherapeutic strategies, these therapies may also provide a more holistic approach to controlling protozoan and helminth infection in humans on both an individual and population scale. In addition, elucidating this complex relationship may also shape future utilization of helminth and/or helminth-derived products as protective or ameliorative agents in immune- and microbial-driven conditions like IBD. Though this area of inquiry is still in its infancy and only growing as we dive deeper into the world of the gut microbiota, studying the host–parasite–microbiota axis may, ultimately, have profound implications for global human health.
